# Green Approach to Develop Bee Pollen-Loaded Alginate Based Nanofibrous Mat

**DOI:** 10.3390/ma14112775

**Published:** 2021-05-24

**Authors:** Ayben Pakolpakçıl, Zbigniew Draczynski

**Affiliations:** Faculty of Material Technologies and Textile Design, Lodz University of Technology, 116 Żeromskiego Street, 90-924 Lodz, Poland; zbigniew.draczynski@p.lodz.pl

**Keywords:** bee pollen, nanofiber, biomaterial, green production, electrospinning

## Abstract

Green electrospun materials are gaining popularity in the quest for a more sustainable environment for human life. Bee pollen (BP) is a valuable apitherapeutic product and has many beneficial features such as antioxidant and antibacterial properties. Alginate is a natural and low-cost polymer. Both natural materials show good compatibility with human tissues for biomedical applications and have no toxic effect on the environment. In this study, bee pollen-loaded sodium alginate and polyvinyl alcohol (SA/PVA) nanofibrous mats were fabricated by the electrospinning technique. The green electrospun nanofibrous mats were analyzed by scanning electron microscopy (SEM), Fourier transforms infrared spectroscopy (FTIR), and differential scanning calorimeter (DSC). According to the findings of the study, the toxin-free electrospinning method is suitable for producing green nanomaterial. Because of the useful properties of the bee pollen and the favorable biocompatibility of the alginate fibers, the bee pollen-loaded SA/PVA electrospun mats have the potential for use in a variety of biomedical applications.

## 1. Introduction

In the past ten years, electrospinning has achieved great interest from the scientific community for its ability to create green micro and nanoscale polymeric fiber that is in high demand due to the use of cheap raw materials, non-toxic solvents, and the effectiveness of the fabrication technology for mass production of these products. According to the Grand View Research Inc. report, the global nanofibers market size was estimated at USD 477.7 million in 2016. Changes in customer and product trends drive the market. It is expected that the manufacture of a wide range of products from composite nanoscale fibers will drive industry innovation and thus increase its penetration in the medical, automotive, and textile markets [1].

Nanofiber structures have superior properties, such as high surface area and adjustable porosity. As a result, the use of these materials has been extensively researched in a variety of fields, including wound dressing, scaffolding, filtering, and packaging. Electrospinning is a simple, inexpensive, and green process capable of generating nanofibers. In the electrospinning process, the polymer solution is extruded by the electric field force from the capillary tube, and a Taylor cone can be formed at the end of the capillary. Positive charges accumulate on the surface of the Taylor cone as the electric field density increases, overcoming the surface tension and causing the spray of fluid. The solvent evaporates quickly, resulting in a continuous ultra-fine polymer fiber [2,3].

Since ancient times, bee products have been used in the medical field. In recent years, many researchers have become interested in developing bionanomaterials using bee products [4]. The use of bee products, such as honey [5,6,7,8,9,10,11,12,13,14,15] and propolis [16,17,18,19,20,21,22,23,24,25], for developing nanofibrous biomedical applications has been studied extensively. Bee pollen is another bee product that may well influence human health. It is used for the health benefits it brings due to its anti-carcinogenic, antioxidant, anti-inflammatory, antibacterial, and anti-allergic properties. Bee pollen is produced by the collection of floral pollen, from flowers and seedlings by stingless bees and honey bees, along with nectar or honey, bee secretions, wax, and enzymes. It consists of carbohydrates, proteins, amino acids, lipids, phenolic compounds, vitamins, and minerals [26,27,28,29,30].

Alginate is one of the most abundant natural polymers in the world. Sodium alginate (SA) is biodegradable, biocompatible and non-toxic, and is obtained from brown algae. Because of its structural similarities to the extracellular matrix, and its gelling properties under conditions compatible with biological activities, it is widely used in biomedical engineering applications, for example, drug delivery, in vitro cell culture, wound healing, and tissue engineering [31,32,33]. Electrospun alginate-based nanofibers have been developed for use in biomedical applications such as wound dressing [34,35,36], tissue engineering [37,38], and drug delivery systems [39,40]. The SA polymer in a water-based solution cannot be electrospun alone due to its inadequate chain entanglements. Polyvinyl alcohol (PVA) or polyethylene oxide (PEO) is usually used to accompany the electrospinning of sodium alginate. PVA is a hydrophilic linear polymer, and it has useful properties such as low toxicity, water-solubility, biocompatibility, and biodegradability [41].

**Figure 1:**
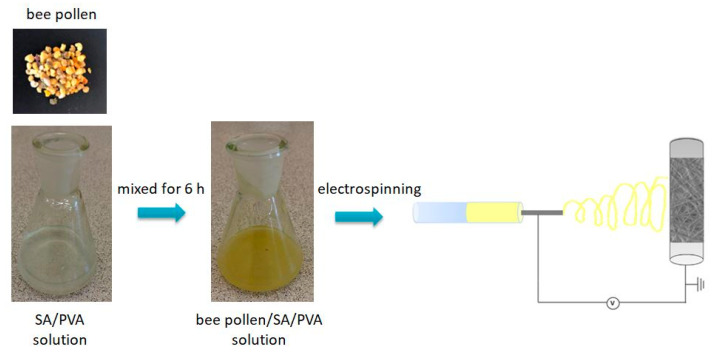
Scheme of the bee pollen-loaded SA/PVA electrospun nanofibrous mats fabrication by electrospinning.

It has been suggested that using a combination of bee pollen and alginate to develop green nanofibrous material could be an alternative method in bioengineering. As yet, there has been no study investigating the development of bee pollen-loaded polymeric nanofiber. In this study, bee pollen-loaded alginate-based nanofibrous mats were fabricated by electrospinning technique (Figure 1). The morphology, chemical composition, and thermal properties of the electrospun mats were investigated. The study results revealed that the bee pollen-loaded alginate-based nanofibrous materials are an environmentally sustainable material using the green electrospinning technique.

## 2. Experimental

### 2.1. Materials

Bee pollen was purchased from the local market (Apipol-Farma Sp. zo.o, Myslenice, Poland). SA polymer was obtained from Cargill Inc. (Minneapolis, MN, USA) and PVA (M_w_ 85,000–124,000 g/mol with 87–89% hydrolysis) polymer was purchased from Sigma Aldrich (Sigma-Aldrich Sp. Zoo, Poznan, Poland). The deionized water (DE 20 Plus-Polna, Przemysl, Poland) was used throughout the experimental study.

### 2.2. Methods

#### 2.2.1. Preparation of Solutions for Electrospinning

Electrospinning polymer solutions were prepared by stirring 10 g PVA in deionized water (100 mL) for 6 h at 90 °C temperature and 2 g SA in deionized water (100 mL) for 4 h at 50 °C temperature. Then, the two polymer solutions were mixed in a 4:1 (volume: volume) ratio. The various concentrations of bee pollen (1, 2, and 3 g) were added to the SA/PVA (100 mL) solutions and mixed for 6 h to get homogeneous solutions.

#### 2.2.2. Electrospinning Process

The nanofibrous mats were produced in an electrospinning device (home-made design, Lodz University of Technology, Lodz, Poland) with the prepared solutions. The SA/PVA and the bee pollen-loaded SA/PVA electrospinning solutions were placed in a plastic syringe tube (ALMO-Erzeugnisse Erwin Burch GmbH, Bad Arolsen, Germany) fed through a metal nozzle of 0.6 mm inner diameter (KD-Fine, KDM^®^ KD Medical GmbH, Berlin, Germany). The nanofibrous mats were attained at a flow rate of 0.8 mL/h with an applied voltage of 25 kV. The nozzle-tip-to-collector distance was fixed at 15 cm. A drum collector at 200 rpm was covered with aluminum foil and samples were collected on it. All solution preparations and electrospinning processes were carried out at room conditions (temperature = 25 ± 2 °C; relative humidity = 50 ± 5%). The samples were named SA/PVA, 1BP-SA/PVA, 2BP-SA/PVA and, 3BP-SA/PVA, respectively.

#### 2.2.3. Measurements and Characterizations

An apparent viscometer (Brookfield Viscometer RV-DV II, AMETEK Brookfield, Middleboro, USA) was used to measure the Brookfield viscosity of the electrospinning solutions at room temperature (25 ± 2 °C) using an SC4-27 spindle at a constant speed of 100 rpm. A conductivity meter (Bonajay Multifunction EC Meter, Bonajay Technology Co., Ltd., Shenzhen, China) was used to determine the conductivity of the electrospun solutions. Three measurements were performed. The mean and standard deviation values of the conductivity and apparent viscosity were calculated.

A scanning electron microscope (Nova™ NanoSEM 230, FEI Company, Hillsboro, OR, USA) was used for morphology observation of the SA/PVA and the bee pollen-loaded SA/PVA nanofibrous mats. Image J software (National Institutes of Health and the Laboratory for Optical and Computational Instrumentation (LOC), Madison, WI, USA) was used to measure the diameter of the fiber. At least 100 measurements were taken from the SEM images of samples. The mean and standard deviation of the measurements were calculated.

The functional groups of the bee pollen and the SA/PVA, and the bee pollen-loaded SA/PVA nanofibrous mats) were analyzed by FTIR-ATR (Attenuated Total Reflectance) spectroscopy (Nicolet 6700, Thermo Electron Corp., Madison, WI, USA). The spectra were obtained by recording samples from a wavelength of 600–4000 cm^−1^. SpectraGryph software was used for investigation (Dr. Friedrich Menges, Oberstdorf, Germany).

Thermal behavior of the SA/PVA and the bee pollen-loaded SA/PVA nanofibrous mats were investigated using a differential scanning calorimeter (DSC Q2000, TA Instruments, New Castle, DE, USA). The electrospun samples were weighed and sealed in aluminum pans. Then, the temperature was elevated from room temperature to 220 °C at a heating rate of 20 °C/min under a nitrogen atmosphere. TA Universal Analysis 2000 software was used for investigation (TA Instruments, New Castle, DE, USA).

## 3. Results

### 3.1. Properties of Electrospinning Solutions

**Figure 2:**
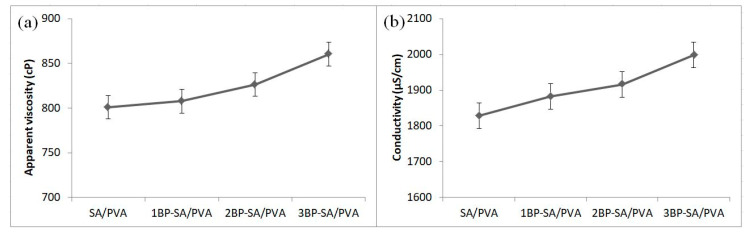
Apparent viscosity (**a**) and conductivity (**b**) of the electrospinning solutions with varying bee pollen content: 0, 1, 2, and 3 weight %.

The process of electrospinning and the morphology of the electrospun nanofibers are influenced by solution parameters. Viscosity and electric conductivity are key factors [2,3]. Thus, before the electrospinning process, these parameters were determined. The electrical conductivity and apparent viscosity of the SA/PVA solution and SA/PVA solutions containing bee pollen are shown in Figure 2. The SA/PVA solution exhibited the lowest apparent viscosity of 801 cP. The addition of bee pollen to the SA/PVA solution increased the electrospinning solutions’ apparent viscosity. The SA/PVA solution with 3 weight % bee pollen concentration exhibited the highest apparent viscosity of 860 cP. The SA/PVA solution had the lowest conductivity at 1828 μS/cm, while the SA/PVA solution with 3 weight % bee pollen had the highest conductivity at 1998 μS/cm.

### 3.2. Morphology of the Nanofibrous Mats

**Figure 3:**
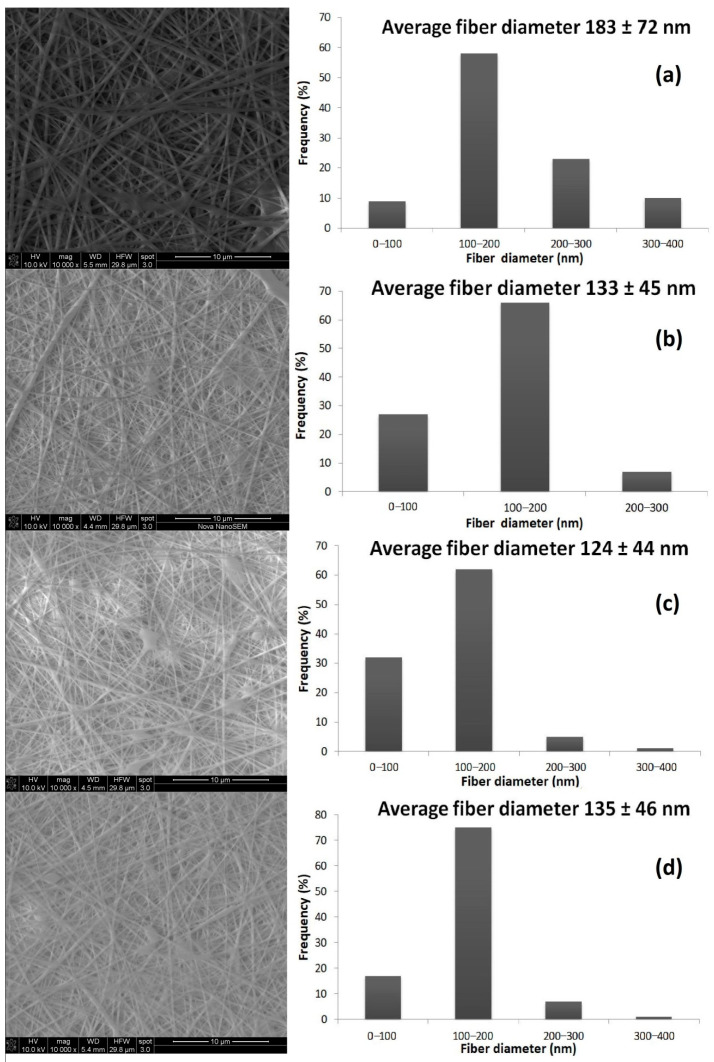
SEM images and fiber diameter distributions of the electrospun SA/PVA fibers obtained from (**a**) SA/PVA, (**b**) 1BP-SA/PVA, (**c**) 2BP-SA/PVA, and (**d**) 3BP-SA/PVA (×10,000).

The morphological images and fiber diameter distributions of the electrospun SA/PVA and bee pollen-loaded SA/PVA nanofibrous mats are shown in Figure 3. SEM images demonstrated that the electrospun SA/PVA nanofibrous mat exhibited randomly oriented fibers with almost the same diameter along their lengths in the strip, and it lacked any beads (Figure 3a). It has been reported that the average fiber diameter of SA/PVA nanofibers is between 150 and 250 nm [42]. In this study, the average diameter of the electrospun SA/PVA nanofiber was 183 nm. This result is similar to the literature. The morphology of the electrospun SA/PVA nanofibrous mats containing different quantities of bee pollen indicated that the randomly oriented fibers had similar diameters along their lengths in the strip, as well as a few beads (Figure 3b–d). The presence of bee pollen in the SA/PVA solution negatively affected the electrospinning process results when comparing sample SEM images (Figure 3b–d). With the presence of bee pollen in the SA/PVA solutions, the average diameter of the nanofibrous mats decreased. The average fiber diameter of the electrospun bee pollen-loaded SA/PVA nanofibrous mats was found to be in the range of 100–150 nm. This may result in increased conductivity of the electrospinning solutions, which causes the jet to stretch more [2,3], resulting in a thinner nanofiber diameter. The image (Figure 3) shows that SA/PVA nanofibrous mats had a smooth and uniform morphology, whereas bee pollen-loaded SA/PVA nanofibrous mats had a less uniform structure.

### 3.3. FTIR Analysis

**Figure 4:**
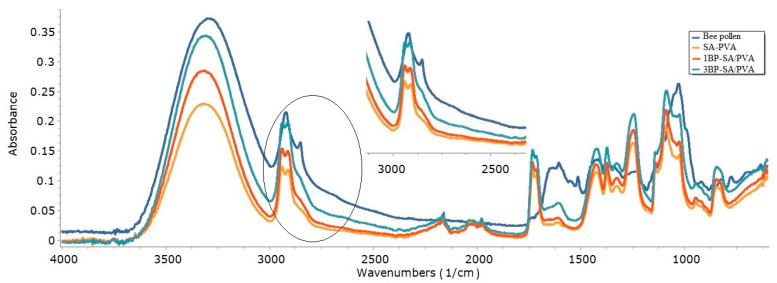
FTIR-ATR spectra of the bee pollen, the electrospun SA/PVA, and bee pollen-loaded SA/PVA nanofibers.

The functional groups of bee pollen, SA/PVA, and bee pollen-loaded SA/PVA nanofibrous mats were determined by FTIR-ATR spectroscopy (Figure 4). The characteristic peaks of the bee pollen were observed at 3300 cm^−1^, 2922 cm^−1^, 1606 cm^−1^, 1514 cm^−1^, and 1028 cm^−1^ were assigned to OH groups, CH stretching, CC stretching, and CO stretching, respectively. The outcome is consistent with the literature [43].

The characteristic peaks of the SA/PVA nanofiber were observed at 3310 cm^−1^, 2941 cm^−1^, 1732 cm^−1^, and 1091 cm^−1^ were assigned to OH groups, CH stretching, and CO stretching, respectively [36,41]. The characteristic peaks of the electrospun bee pollen-loaded SA/PVA nanofibers (1BP-SA/PVA and 3BP-SA/PVA) were observed at 3310 cm^−1^, 2939 cm^−1^, 1732 cm^−1^, and 1088 cm^−1^ were assigned to OH groups, CH stretching and CO stretching, respectively. As a result of CH stretching vibrations of different groups in bee pollen, the adsorption bands in the frequency range of 2880–2860 cm^−1^ were broadened with the addition of bee pollen. Furthermore, the intensity of the absorption band resulting from the stretching of –OH groups increased, indicating that bee pollen hydroxyl groups were incorporated into the SA/PVA electrospun mat. While the maximum intensity of the hydroxyl of SA/PVA nanofiber was around 0.22, the maximum intensity of hydroxyl increased from around 0.29 to 0.32 as the nanofibers bee pollen concentration increased from 1% to 3%.

### 3.4. DSC Analysis

**Figure 5:**
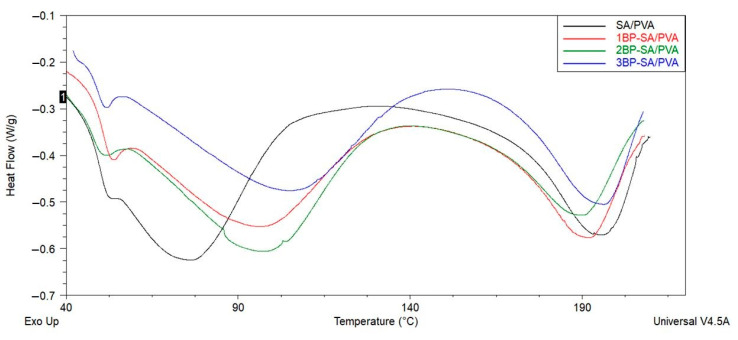
DSC thermograms of the electrospun SA/PVA and bee pollen-loaded SA/PVA nanofibers.

The thermal properties of the electrospun nanofibers were examined by differential scanning calorimeter. Glass transition and melting temperatures are two critical phenomena in polymers that are influenced by the materials’ processing conditions and additives. DSC analysis provides important information about phase transitions within materials [44]. The DSC thermograms of the electrospun SA/PVA and bee pollen-loaded SA/PVA nanofibers are presented in Figure 5. Three endothermic peaks were observed on the DSC curve of the SA/PVA as well as bee pollen-laded SA/PVA. The first relaxation observed at 50–60 °C, was due to relaxation in amorphous regions of PVA [44,45]. The second relaxation in the temperature range of 75–120 °C was caused by the glass transition temperature of the water evaporation [44,45] of PVA, and the third peak starting from 190℃ was due to the melting temperature of the PVA. These results are consistent with the literature [44,45,46]. The glass transition temperature (T_g_) of the SA/PVA, 1BP-SA/PVA, 2BP-SA/PVA, and 3BP-SA/PVA nanofiber samples was determined to be around 76 °C, 95 °C, 101 °C, and 105 °C, respectively. When bee pollen was incorporated into the SA/PVA nanofiber, there was no sharp peak in the melting temperature of the sample. The melting temperature (T_m_) of the SA/PVA, 1BP-SA/PVA, 2BP-SA/PVA, and 3BP-SA/PVA nanofiber samples was determined to be around 196 °C, 192 °C, 190 °C, and 196 °C, respectively.

## 4. Discussion

Water was used as the solvent for the bee pollen, SA, and PVA polymers when preparing the electrospinning solutions. During the experiment, three different bee pollen-containing SA/PVA solutions were prepared. This demonstrated that bee pollen can be encapsulated into alginate-based nanofibers without the use of any harmful solvents. The approach has no negative consequences for human health and is also eco-friendly.

In general, the viscosity of the solution increased as the bee pollen concentrations increased. The increase in viscosity of the electrospinning solutions may be due to hydrogen bonding interactions between bee pollen and SA/PVA polymer mixtures. The presence of bee pollen in the SA/PVA solution increased the conductivity of the solution. Bee pollen contains micronutrients (iron, copper, zinc, manganese, silicon, and selenium) and macronutrients (calcium, phosphorus, magnesium, sodium, and potassium) [26,30]. Therefore this could also lead to an increase in the total quantity of ions in the solution.

To investigate the effect of the electrospinning solution composition on the structure of the nanofiber and electrospinning process, various solutions containing different amounts of bee pollen were prepared and used in the development of nanofiber. SEM images showed that the surface of the electrospun bee pollen-loaded SA/PVA nanofiber had a few defective structures. During the electrospinning process, it was observed although fairly smooth surfaces were produced at concentrations of 2 and 3 weight % bee pollen, the obtained surfaces could not be properly stripped from the aluminum foil. This is thought to be due to the higher sugar content [28,30] as the stickiness increased. Generally, the electrospun bee pollen-loaded SA/PVA nanofibers demonstrated a few imperfections with an average diameter of about 100 nm. The tiny fiber and the highly porous structure of nanofiber is an advantage in biomedical applications, such as wound dressings and scaffolding. Because of these features, nanofibers can mimic the extracellular matrix (like collagen structure, scaffolds are composed of fibers with a diameter in the range of 50–500 nm) of normal skin or tissue and help promote cell growth and spread [31,32].

The FTIR-ATR spectra of the electrospun SA/PVA nanofibers changed after the addition of bee pollen, and the adsorption bands in the frequency range of 2880–2860 cm^−1^ were broadened due to CH stretching vibrations of different groups in bee pollen. The peak intensity of the stretching of –OH groups that occurs depending on the bee pollen concentration indicates the presence of bee pollen in the developed nanofibrous material.

An increase in the glass transition temperature (T_g_) and peak broadening indicated that the ordered association of the SA/PVA molecules was enhanced by the presence of bee pollen. Various factors can affect the glass transition temperature of the material, such as molecular structure and polar groups. It can be assumed that bee pollen decreased the segmental mobility of the polymer, thus inflexible groups increased Tg of nanofibrous material. Bee pollen molecules contain carboxyl groups [47,48] and PVA molecules contain hydroxyl groups [36,41]. It is thought that hydrogen bonds are formed between these two groups. The formation of hydrogen bonds between bee pollen and SA/PVA causes improvement in the compactness and regularity of polymer chains. The thermal properties of the polymeric material depend on the mobility and compactness of the polymer chains and their crystalline structure in the material [49,50]. A minor change was observed in the melting temperature. This result showed that the presence of bee pollen in the SA/PVA nanofibrous mat did not significantly affect the melting temperature of samples.

Alginate has excellent biocompatibility, biodegradability, non-toxicity, gel-forming performance, and is easy to process [31,32]. Previous studies have indicated that bee pollen possesses antimicrobial, antifungal, antiviral, antioxidant, anti-inflammatory, immunostimulating, and local analgesic features [51,52]. Therefore, the bee pollen-loaded SA/PVA mats may have the potential to be used in several biomedical applications due to the beneficial properties of bee pollen and the desirable biocompatibility of SA and PVA polymers. The bee pollen-loaded SA/PVA electrospun mat is easy to fabricate and inexpensive, thanks to the use of an environmentally friendly nanotechnological production method. To the best of our knowledge, in our effort to develop a biomaterial using green electrospinning, we prepared a nanofibrous material based on SA/PVA loaded with bee pollen, which was produced for the first time. However, this study also showed that more research and work need to be done. This report is expected to play a leading role in future studies on this issue.

## 5. Conclusions

This work aimed to be the first approach to the use of bee pollen to obtain nanofibrous material by the electrospinning technique using a cheap, simple, and eco-friendly methodology. The study demonstrates the process and the characterization of the electrospun bee pollen-loaded alginate-based mat. The SEM images of the electrospun bee pollen-loaded alginate-based mats were mostly on a nanometer scale with the presence of a few defects. The incorporation of bee pollen into the SA/PVA electrospun mat was demonstrated by the FTIR spectrum. The DSC thermogram demonstrated that adding bee pollen into the SA/PVA polymer solution caused a remarkable change in the glass transition temperature but it had no significant effect on the melting temperature. The method for green electrospinning of water-based systems may be an alternative way to produce bee pollen-loaded alginate-based nanofibrous material. This study demonstrates how environmentally friendly nanofibrous materials can be designed for use in a wide range of fields, including biomedical applications such as, wound dressings and scaffoldings.

## Data Availability

Not applicable.
